# High-temperature optical properties of indium tin oxide thin-films

**DOI:** 10.1038/s41598-020-69463-4

**Published:** 2020-07-27

**Authors:** Jiwoong Kim, Sujan Shrestha, Maryam Souri, John G. Connell, Sungkyun Park, Ambrose Seo

**Affiliations:** 1 Department of Physics and Astronomy, University of Kentucky, Lexington, KY 40506 USA; 2 Department of Physics, Pusan National University, Busan, 46241 Korea

**Keywords:** Electronic devices, Surfaces, interfaces and thin films, Electronic properties and materials, Phase transitions and critical phenomena

## Abstract

Indium tin oxide (ITO) is one of the most widely used transparent conductors in optoelectronic device applications. We investigated the optical properties of ITO thin films at high temperatures up to 800 °C using spectroscopic ellipsometry. As temperature increases, amorphous ITO thin films undergo a phase transition at ~ 200 °C and develop polycrystalline phases with increased optical gap energies. The optical gap energies of both polycrystalline and epitaxial ITO thin films decrease with increasing temperature due to electron–phonon interactions. Depending on the background oxygen partial pressure, however, we observed that the optical gap energies exhibit reversible changes, implying that the oxidation and reduction processes occur vigorously due to the low oxidation and reduction potential energies of the ITO thin films at high temperatures. This result suggests that the electronic structure of ITO thin films strongly depends on temperature and oxygen partial pressure while they remain optically transparent, i.e., optical gap energies > 3.6 eV.

## Introduction

Tin-doped indium oxide or indium tin oxide (ITO) is a transparent conductor, which is widely used in modern optoelectronic devices such as thin-film transistors, resistive switching memories, and solar cells^1–5^. However, it is mostly unexplored if its high electrical conductivity and optical transparency will remain intact at extreme conditions such as high temperature or pressure. The question concerning the high-temperature property of materials is particularly important as modern technology evolves to device applications and operations in harsh environments above 500 °C^6–8^. Since the bandgap energies of semiconductors usually decrease with increasing temperature^9,10^, we may expect that ITO will also lose its optical transparency at high temperatures. Indeed, indium oxide (In_2_O_3_) single crystals have been reported to show a more significant reduction of its optical gap at high temperatures than semiconductor crystals such as Si and GaAs^11^. This somewhat pessimistic result is understood by a strong interaction between electrons and lattice vibrations (i.e., electron–phonon interaction), affecting the bandgap energy of In_2_O_3_^12^. However, it is essential to note that high concentrations of doped Sn ions and oxygen vacancies play a crucial role in the electronic band structure of ITO and its optical properties. For example, the optical gap energy of ITO is larger than that of undoped In_2_O_3_ because of the existence of free electron carriers^13^, according to the Burstein-Moss effect^14^, and the temperature dependence of free carrier concentration is nearly constant^15^. Hence, investigating the high-temperature optical properties of ITO requires systematic measurements and understanding of the role of dopants and defects as well as electron–phonon interactions.

In this paper, we report the high-temperature optical properties of ITO thin films up to 800 °C. We studied ITO thin films of amorphous (*a*-ITO), polycrystalline (*poly*-ITO), and epitaxial (*epi*-ITO) phases rather than ITO bulk crystals because these thin-film forms are the ones used in various optoelectronic device applications. While *a*-ITO thin films show an abrupt increase of optical gap energies due to crystallization at ~ 200 °C, the optical gap energies of *poly*-ITO and *epi*-ITO thin films decrease gradually as temperature increases. Nevertheless, the optical gap energies (*E*_g_) remain higher than the visible photon energies (i.e., *E*_g_ > 3 eV), meaning that their optical transparency is unaffected. We also found that background oxygen partial pressure at high temperatures alters the optical gap energies of ITO thin films *reversibly* by oxidation and reduction processes. Our results demonstrate that an unintentional oxygen annealing effect at high temperatures can result in different optical and transport properties of ITO thin films.

## Results and discussion

### Amorphous and polycrystalline ITO thin films

**Figure 1:**
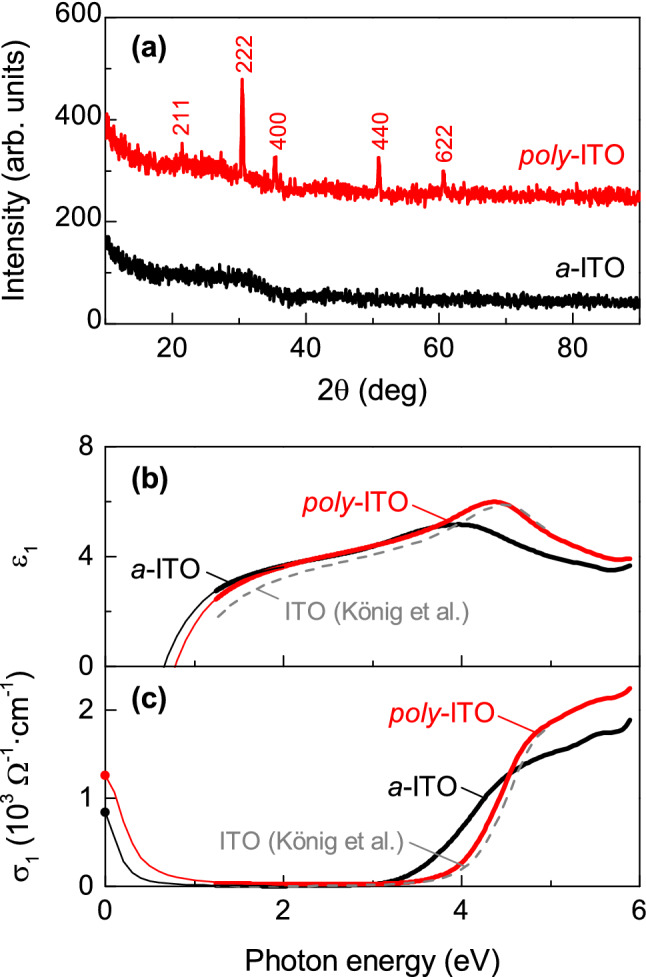
Structural and optical characterization of *a*-ITO and *poly*-ITO thin films. (**a**) XRD *θ*–2*θ* scans of *a*-ITO (black) and *poly*-ITO (red) thin films. According to the diffraction indices of *poly*-ITO, it is a cubic bixbyite structure (ICSD #50848). (**b**) Dielectric constant (*ε*_1_) and (**c**) optical conductivity (*σ*_1_) spectra of *a*-ITO (black) and *poly*-ITO (red) thin films measured at room temperature. The grey dashed lines are the spectra from Ref.^33^ for comparison. The dots at zero photon energy are from *dc*-conductivity measurements. The solid thin lines between 0 and 1.2 eV are interpolated using the Drude model.

We observed that *a*-ITO thin films undergo a phase transition in the annealing process and develop a polycrystalline phase with increased *E*_g_ at high temperatures. Figure 1a shows X-ray diffraction (XRD) *θ*–2*θ* scans of both as-grown *a*-ITO and *poly*-ITO, which are ITO thin films on glass substrates before and after post-annealing at 500 °C, respectively. While the *a*-ITO thin film shows no visible peak, which verifies that the thin film is amorphous, clear diffraction peaks appear in the annealed sample. The diffraction peaks of the *poly*-ITO thin film match well with the cubic bixbyite ITO structure (ICSD #50848). Note that *poly*-ITO thin films exhibit a larger *E*_g_ (i.e., 4.25 ± 0.01 eV) than *a*-ITO thin films (i.e., 3.85 ± 0.01 eV) at room temperature, as shown in Fig. 1b,c. The relation between the complex dielectric function ($\tilde{\varepsilon } = \tilde{n}^{2} = \varepsilon_{1} + i\varepsilon_{2}$), and the optical conductivity ($\sigma_{1}$) is $\sigma_{1} = \frac{{\varepsilon_{2} \omega }}{4\pi }$ , where *ω* is the photon energy. The spectral shapes of $\varepsilon_{1}$ and $\sigma_{1}$ and the change of *E*_g_ are consistent with each other according to the Kramers–Kronig relation. We extrapolated the spectra below 1.2 eV using the Drude model^16^ to match with the *dc* conductivities (i.e., $\sigma_{1}$ at 0 eV) of these samples. The estimated plasma frequencies, where the dielectric constant crosses zero, increase from 0.7 ± 0.1 eV (*a*-ITO) to 0.8 ± 0.1 eV (*poly*-ITO). Since the plasma frequency (*ω*_p_) is proportional to the carrier concentration (*n*_*e*_) ($\omega_{{\text{p}}}^{2} = 4\pi n_{e} e^{2} /m$, where *e* and *m* are the elementary charge and the effective mass of electron, respectively), the enhanced *E*_g_ in *poly*-ITO thin films is presumably due to increased free carriers (i.e., the Burstein-Moss effect^14^) and reduced disorder^17^.

**Figure 2:**
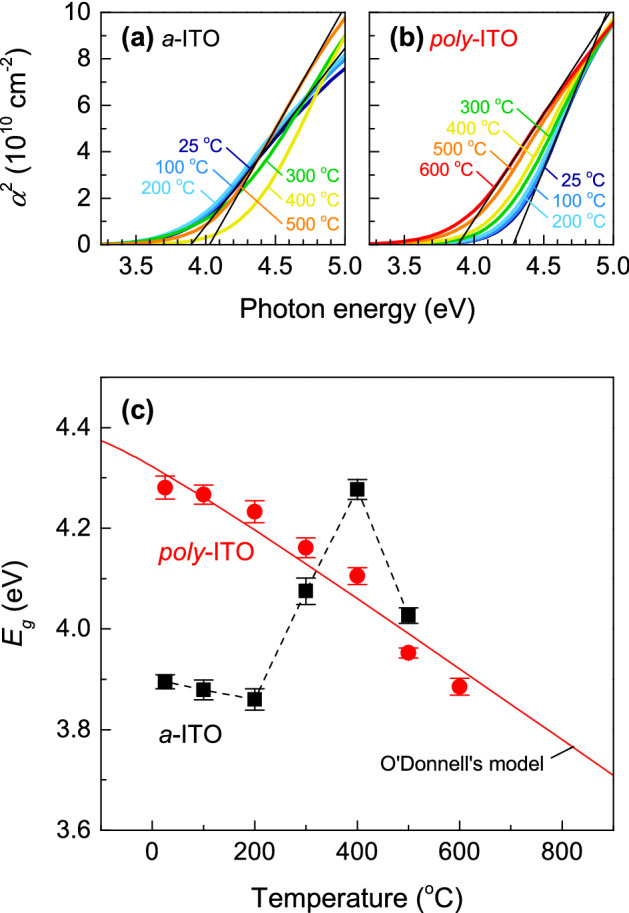
High-temperature optical properties of *a*-ITO and *poly*-ITO thin films. Absorption coefficient squared (*α*^2^) vs. photon energy plots for (**a**) *a*-ITO and (**b**) *poly*-ITO thin films obtained from ellipsometry spectra at high temperatures up to 500 °C and 600 °C, respectively. The black lines are the linear extrapolation of *α*^2^ spectra at 25 °C and 500 °C (600 °C) for the *a*-ITO (*poly*-ITO) thin film. (**c**) Optical gap energies of *a*-ITO and *poly*-ITO thin films as a function of temperature. The solid red line is a curve fit from O’Donnell’s model^10^.

To determine high-temperature *E*_g_ of *a*-ITO and *poly*-ITO thin films, we calculated the absorption coefficient (*α*) from the extinction coefficient (*k*) using *α* = 4*πk*/*λ*, where *λ* is the wavelength of the photon. Since the direct *E*_g_ has the relation: *α *≈ (*ω *– *E*_g_)^1/2^ (Ref.^14^), Figure 2a,b shows *α*^2^ vs. photon energy plots of *a*-ITO and *poly*-ITO thin films above room temperature, respectively. *E*_g_ is estimated by linear extrapolation at each temperature. Figure 2c shows *E*_g_ as a function of temperature for both *a*-ITO and *poly*-ITO thin films. Note that *E*_g_ of *a*-ITO thin films shows an abrupt change above 200 °C, where polycrystalline ITO starts forming, as reported in Refs.^18,19^. On the other hand, *E*_g_ of *poly*-ITO thin films decreases gradually with increasing temperature, as also shown in Fig. 2c. We suggest that strong electron–phonon interaction of *poly*-ITO thin films is responsible for the temperature dependence of *E*_g_. According to O’Donnell’s model^10^, *E*_g_ at absolute temperature *T* is described by

1 $$E_{g} (T) = E_{g} (0) - S\langle\hbar \omega_{ph}\rangle \left[ {\coth \left( {\frac{{\langle\hbar \omega_{ph}\rangle }}{{2k_{B} T}}} \right) - 1} \right],$$

where *S* is a dimensionless coupling constant, $\langle\hbar \omega_{ph}\rangle$ is the average phonon energy, and *k*_*B*_ is the Boltzmann constant. Using the average phonon energy of In_2_O_3_ (38.6 meV)^11^, we can obtain *E*_g_(0) = 4.40 ± 0.04 eV and *S* = 4.2 ± 0.5, as shown in Fig. 2c (red line). Note that the coupling constant *S* is smaller than that of undoped In_2_O_3_ (*S* = 8.24)^11^, implying that lattice imperfections such as substitutional Sn ions, oxygen vacancies, and grain boundaries, at which phonons are scattered^20^, suppress the electron–phonon interactions in *poly*-ITO thin films.

### Epitaxial ITO thin films

**Figure 3:**
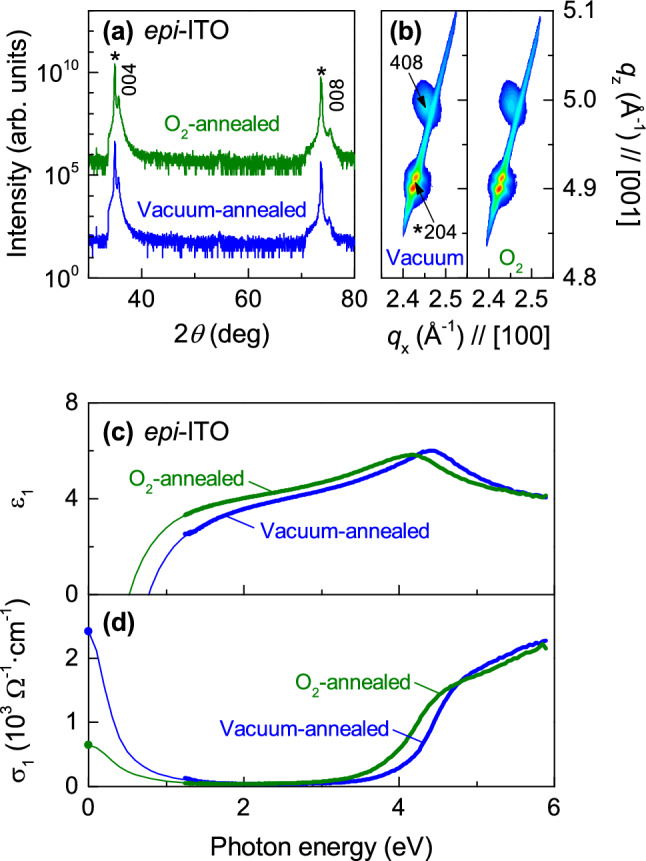
Structural and optical characterization of *epi*-ITO thin films. (**a**) XRD *θ*-2*θ* scans of *epi*-ITO thin films annealed in O_2_ (green) and vacuum (blue) conditions. The asterisks (*) and the indices denote the peaks from the YSZ substrate and cubic bixbyite ITO thin films, respectively. (**b**) X-ray reciprocal space maps near the 204-reflection of YSZ. Both samples show clear 408-reflections from *epi*-ITO thin films. (**c**) *ε*_1_ and (**d**) *σ*_1_ spectra of vacuum-annealed and O_2_-annealed *epi*-ITO thin films at room temperature. The dots at zero photon energy are from *dc*-conductivity measurements. The solid thin lines between 0 and 1.2 eV are interpolated using the Drude model.

To explore intrinsic high-temperature optical properties, we measured *epi*-ITO thin films prepared in two different annealing conditions, i.e., *p*(O_2_) = 10 mTorr (O_2_-annealed) and *p*(O_2_) < 1.0 × 10^–6^ Torr (vacuum-annealed). Figure 3a,b shows XRD *θ*-2*θ* scans and reciprocal space mappings, respectively. Both *epi*-ITO thin films are coherently grown without any notable secondary phases. It is noteworthy that these samples show identical in-plane (10.25 ± 0.01 Å) and out-of-plane lattice constants (10.07 ± 0.01 Å) regardless of the annealing conditions. The *epi*-ITO thin films are under tensile strain (~ 1.3%) due to the lattice mismatch with YSZ substrates^21^. However, the room-temperature optical spectra of *epi*-ITO thin films strongly depend on the annealing conditions presumably due to different concentrations of oxygen vacancies. It was found that the carrier concentration of In_2_O_3_ thin films decreased as increasing the O_2_ pressure during growth^22,23^. DFT calculations suggested that the oxygen vacancies are shallow donors in In_2_O_3_, contributing to the carrier concentration^24,25^. Figure 3c shows *ε*_1_ and $\sigma_{1}$ spectra of *epi*-ITO thin films, respectively. The vacuum-annealed *epi*-ITO thin film shows both increased *ω*_p_ (0.8 ± 0.1 eV) and *E*_g_ (i.e., 4.26 ± 0.03 eV) compared to the O_2_-annealed *epi*-ITO thin film (*ω*_p_ = 0.5 ± 0.1 eV and *E*_g_ = 4.00 ± 0.05 eV). This observation is consistent with the Burstein–Moss effect^14^ since larger free carrier concentrations result in higher *E*_g_ values. Therefore, the annealing conditions influence the number of oxygen vacancies, which are electrons donors^13^, and increase (decrease) the carrier concentrations by reducing (oxidizing) *epi*-ITO thin films and change their optical properties without any noticeable difference in the crystal structure.

**Figure 4:**
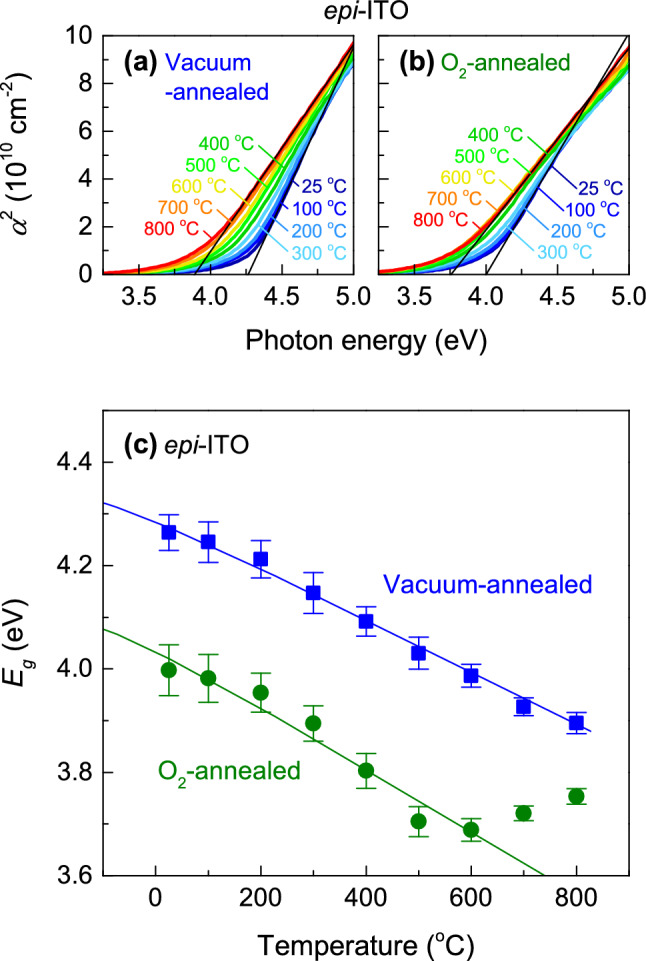
High-temperature optical properties of *epi*-ITO thin films. *α*^2^ vs. photon energy plots for (**a**) vacuum-annealed and (**b**) O_2_-annealed *epi*-ITO thin films obtained from ellipsometry spectra at high temperatures up to 800 °C. The black lines show the linear extrapolation of *α*^2^ spectra at 25 °C and 800 °C. (**c**) Optical gap energies of vacuum-annealed and O_2_-annealed *epi*-ITO thin films as a function of temperature. The blue and green lines are curve fits of vacuum-annealed and O_2_-annealed *epi*-ITO thin films, respectively, using O’Donnell’s model^10^.

We measured *E*_g_ of *epi*-ITO thin films at high temperatures while maintaining the same environmental conditions as the post-annealing process. Figure 4a,b shows *α*^2^ vs. photon energy plots for vacuum-annealed and O_2_-annealed *epi*-ITO thin films, respectively. Figure 4c shows *E*_g_ of *epi*-ITO thin films as a function of temperature obtained from the spectra. Note that both samples show systematically decreasing *E*_g_ with increasing temperature. Based on the average phonon energy of In_2_O_3_ (38.6 meV)^11^, we find *E*_g_(0) = 4.33 ± 0.01 eV and *S* = 3.0 ± 0.1 for the vacuum-annealed *epi*-ITO thin film and *E*_g_(0) = 4.10 ± 0.02 eV and *S* = 3.6 ± 0.3 for the O_2_-annealed *epi*-ITO thin film. Interestingly, the O_2_-annealed *epi*-ITO shows an almost constant *E*_g_ above 600 °C. We speculate that these steady *E*_g_ values above 600 °C are related to the phase transition of In_2_O_3_ from a cubic bixbyite structure to a rhombohedral corundum structure^26^ because the *E*_g_ of corundum phase (3.8 eV)^27^ is higher than bixbyite phase (2.7 eV)^11^ at room temperature. However, it should be checked by high-temperature structural characterizations in future studies.

### Oxidation and reduction of ITO thin films at high temperatures

**Figure 5:**
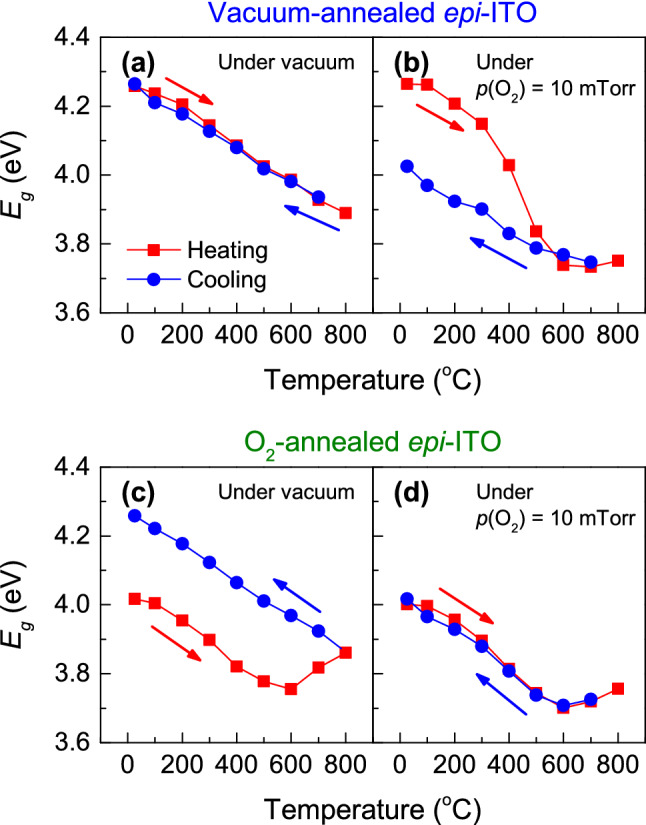
Optical gap energies of *epi*-ITO thin films during oxidation and reduction processes. The vacuum-annealed *epi*-ITO thin film is exposed to (**a**) vacuum and (**b**) *p*(O_2_) = 10 mTorr during the thermal cycle. The O_2_-annealed *epi*-ITO thin film is exposed to (**c**) vacuum and (**d**) *p*(O_2_) = 10 mTorr during the thermal cycle. The red and blue arrows indicate the directions of heating and cooling processes, respectively.

We observed that *reversible* oxidation and reduction processes affect *E*_g_ of *epi*-ITO thin films at high temperatures. Figure 5 shows temperature-dependent *E*_g_ of vacuum-annealed or O_2_-annealed *epi*-ITO thin films measured under the conditions of vacuum (*p*(O_2_) < 1.0 × 10^–6^ Torr) or oxygen (*p*(O_2_) = 10 mTorr). When the vacuum-annealed *epi*-ITO thin film is placed under the vacuum environment (Fig. 5a), its *E*_g_ shows identical temperature-dependence during the heating and cooling cycles. However, when the sample is under the oxygen environment (Fig. 5b), its *E*_g_ decreases significantly at around 300 °C during the heating process, and its *E*_g_ becomes about 0.23 eV smaller than the initial *E*_g_ after cooling down to room temperature. This observation indicates that exposure to high temperatures above 300 °C oxidizes *epi*-ITO thin films and reduces free electron carriers.

In contrast, when the O_2_-annealed *epi*-ITO thin film is located under the vacuum condition, its *E*_g_ at room temperature increases about 0.24 eV after the heating and cooling cycle (Fig. 5c) whereas its *E*_g_ does not change after the thermal cycling under the O_2_ environment (Fig. 5d). The increased *E*_g_ under the vacuum condition reveals that oxygen vacancies and free-electron carriers are generated at high temperatures. Therefore, we can see that the background oxygen partial pressure at high temperatures affect the number of oxygen vacancies, which alters *E*_g_ of *epi*-ITO thin films. Note that the change of *E*_g_ is reversible (compare Fig. 5b with Fig. 5c), meaning that the oxidation and reduction potential energies of the ITO thin films are so low that oxidation and reduction processes occur vigorously at high temperatures.

## Conclusion

We have investigated the optical properties of ITO thin films at high temperatures using SE. The *a*-ITO thin films undergo a phase transition from amorphous to polycrystalline above 200 °C, and its *E*_g_ increases during the transition due to increased free carriers and reduced disorder. The *poly*-ITO and *epi*-ITO thin films exhibit a decrease in *E*_g_ with increasing temperature due to electron–phonon interactions. By monitoring *E*_g_ of *epi*-ITO thin films, we have observed that the oxidation and reduction processes occur reversibly at high temperatures depending on background oxygen partial pressure. All ITO thin films exhibit *E*_g_ higher than 3.5 eV up to 800 °C despite various changes in their electronic structures, suggesting that ITO thin films possess robust optical transparency for high-temperature device applications.

## Methods

We synthesized *a*-ITO and *poly*-ITO thin films grown on glass substrates. The *a*-ITO thin films were grown by using the *dc*-magnetron sputtering technique with a mixture gas of Ar and O_2_. The as-grown *a*-ITO thin films were crystallized into *poly*-ITO thin films after annealing at 500 °C under O_2_ environment (*p*(O_2_) = 10 mTorr). We fabricated the *epi*-ITO thin films on yttria-stabilized zirconia (YSZ) Y:ZrO_2_ (001) substrates using pulsed laser deposition (PLD). The PLD conditions were the oxygen partial pressure (*p*(O_2_)) of 10 mTorr, and the laser (KrF excimer laser (*λ* = 248 nm)) influence of 1.5 J/cm^2^ with the repetition rate of 10 Hz. The substrate temperature was 600 °C to grow high crystalline *epi*-ITO thin film^28^. After the *epi*-ITO thin film growth, the samples were annealed under vacuum (*p*(O_2_) < 1.0 × 10^–6^ Torr) and O_2_ (*p*(O_2_) = 10 mTorr) environments, respectively, to control oxygen vacancies inside the thin films. Table S1 summarizes the growth condition of various ITO thin films in this study. We performed optical spectroscopic measurements with photon energies from 1.2 to 6.0 eV using a spectroscopic ellipsometer (Woollam M-2000) equipped with a custom-built vacuum chamber^29^ with an incident angle of 65° at high temperatures. We measured *a*-ITO (*poly*-ITO) thin films at *p*(O_2_) = 10 mTorr up to 500 °C (600 °C) before the glass substrate is deformed^30^. For *epi*-ITO thin films, we measured up to 800 °C under vacuum (*p*(O_2_) < 1.0 × 10^–6^ Torr) and oxygen (*p*(O_2_) = 10 mTorr) environments. We extracted the complex refractive index, $\tilde{n} = n + ik$, where *n* and *k* are the refractive index and the extinction coefficient, respectively, from the measured spectroscopic ellipsometry parameters, Ψ and Δ, which are the amplitude ratio and phase difference^31^, using numerical interaction process based on the Fresnel equation^32^. (See the Supplementary material for details.) We used a single slab model, i.e., air/ITO/YSZ, without any correction of surface roughness.

## Supplementary information

Supplementary Information.
