# Supplementary material for: High-temperature optical properties of indium tin oxide thin-films
Source: Sci Rep. 2020 Jul 27;10:12486. doi: 10.1038/s41598-020-69463-4 (PMC7385179; doi:10.1038/s41598-020-69463-4)
Supplement: Supplementary file 1 — Supplementary Information. [file 41598_2020_69463_MOESM1_ESM.pdf]

[Supplemental Material]

High-temperature optical properties of indium tin oxide thin-films

Jiwoong Kim,[1,2] Sujan Shrestha,[1] Maryam Souri,[1] John G. Connell,[1] Sungkyun Park,[2] Ambrose Seo[1]

[1]*Department of Physics and Astronomy, University of Kentucky, Lexington, KY 40506, USA*

[2]*Department of Physics, Pusan National University, Busan 46241, Korea*

This file includes:

Figures S1-S5

Tables S1

Elimination of backside reflection for spectroscopic ellipsometry

We roughened the backside of glass substrates by using sandpaper (Fig. S1) to avoid the backside reflection in the spectroscopic ellipsometry (SE) spectra[1] of the *a*-ITO and *poly*-ITO thin-films. We grew the *epi*-ITO thin-films on one-side polished YSZ substrates for the same reason. As a result, the effect of backside reflection is eliminated in the SE spectra.

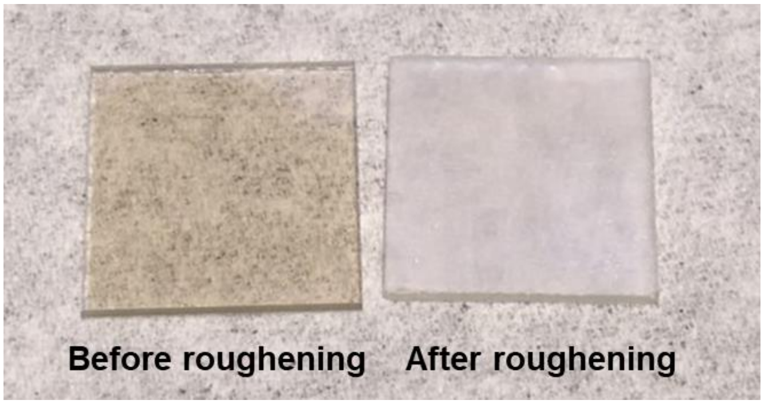

Figure S1. A photograph of ITO thin films grown on glass substrates. As-grown ITO thin films are transparent because of the high transparency of the ITO thin film and the glass substrate (left). For the optical spectroscopic ellipsometry measurements, we have prepared samples by roughening the backside of the substrates using sandpaper (right).

Surface roughness of ITO thin-films

The surface topography of ITO thin films was observed by atomic force microscopy (AFM) (Fig. S2). The root-mean-square (RMS) roughness values of the *a*-ITO, *poly*-ITO, vacuum-annealed *epi*-ITO, and O_2-annealed *epi*-ITO thin films are 1.9 nm, 0.3 nm, 1.5 nm, and 1.1 nm, respectively. The RMS roughness is much smaller than the thickness of the ITO thin-films (150 nm for the *a*-ITO and *poly*-ITO thin films and 50 nm for the *epi*-ITO thin-films), confirming that these samples are suitable for SE measurements and simulations. Therefore, we used a single slab model (air/roughness layer/ITO/substrate) with the surface roughness less than 2 nm.

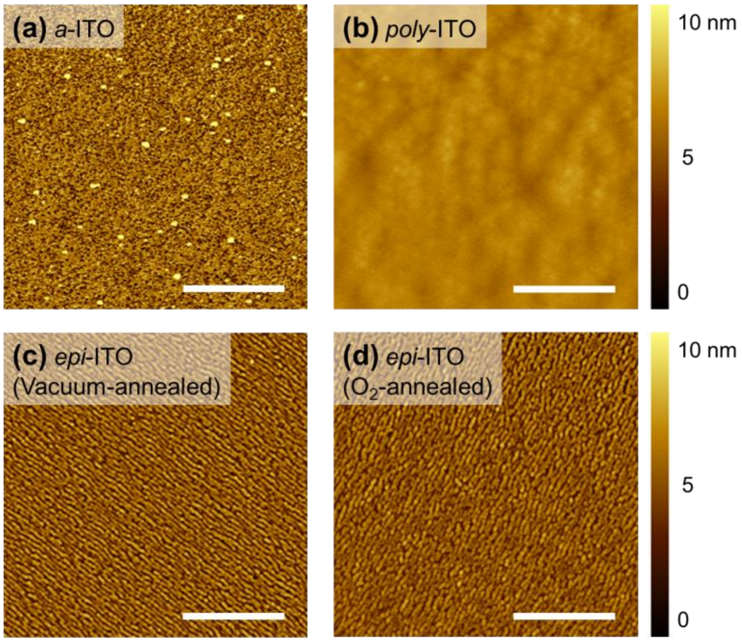

Figure S2. The AFM topography images of (a) *a*-ITO, (b) *poly*-ITO, and *epi*-ITO samples after annealing in (c) vacuum and (d) O_2 environments. The scale bar is 1 μm.

Complex refractive index spectra of substrates

Before determining the SE spectra of the ITO thin-films at high temperatures, we obtained the complex refractive index spectra of glass and YSZ (001) substrates at high temperatures (Fig. S3). We used the corresponding results to extract the optical constants of ITO thin films at high temperatures as the substrate layer in the SE simulations.

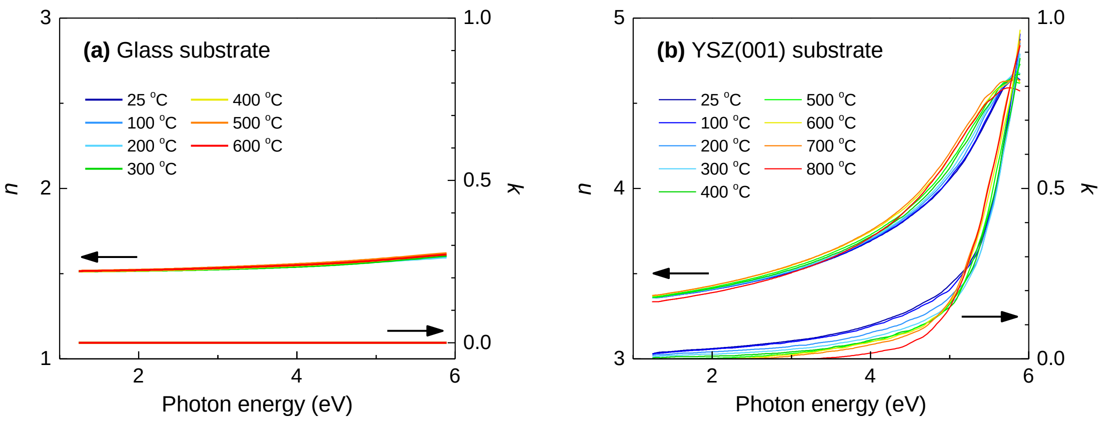

Figure S3. The refractive index (*n*) and extinction coefficient (*k*) spectra of (a) glass and (b) YSZ (001) substrates obtained from the SE spectra measured at high temperatures.

Spectroscopic ellipsometry spectra of ITO thin films with fitting results

The SE spectra of ITO thin-films were fitted by single slab models with surface roughness for *a*-ITO thin films (air/roughness layer/ITO/glass) and without surface roughness (air/ITO/glass or air/ITO/YSZ) for *poly*-ITO and *epi*-ITO thin films. The thickness of the roughness layer of *a*-ITO, estimated by the Bruggeman effective medium approximation, is about 2 nm (Ref. 2). Figure S4 shows the experimental data at room temperature with the fitting results, representing the excellent curve fits.

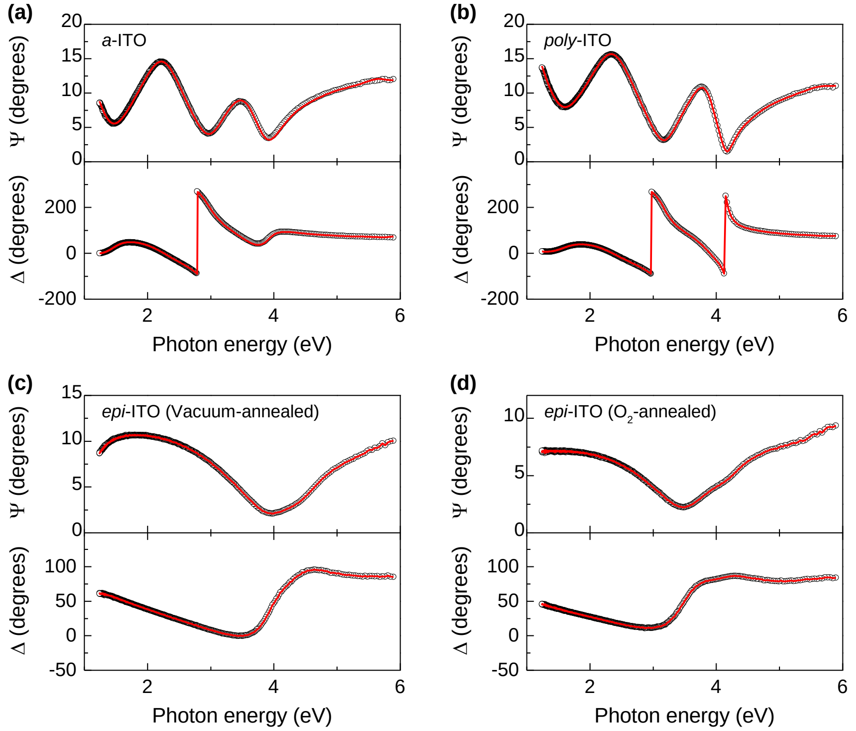

Figure S4. The SE spectra of (a) *a*-ITO, (b) *poly*-ITO, and *epi*-ITO after annealing in (c) vacuum and (d) O_2 environments measured at room temperature (25 ºC). The solid red lines are curve fits using a single slab model.

Complex refractive index spectra of amorphous, polycrystalline and epitaxial ITO thin films at high temperatures

The complex refractive index spectra of ITO thin-films were obtained by the numerical iteration process using a single slab model (Fig. S5). We used the complex refractive index spectra of glass and YSZ (001) substrates (Fig. S3) as substrate data in the model.

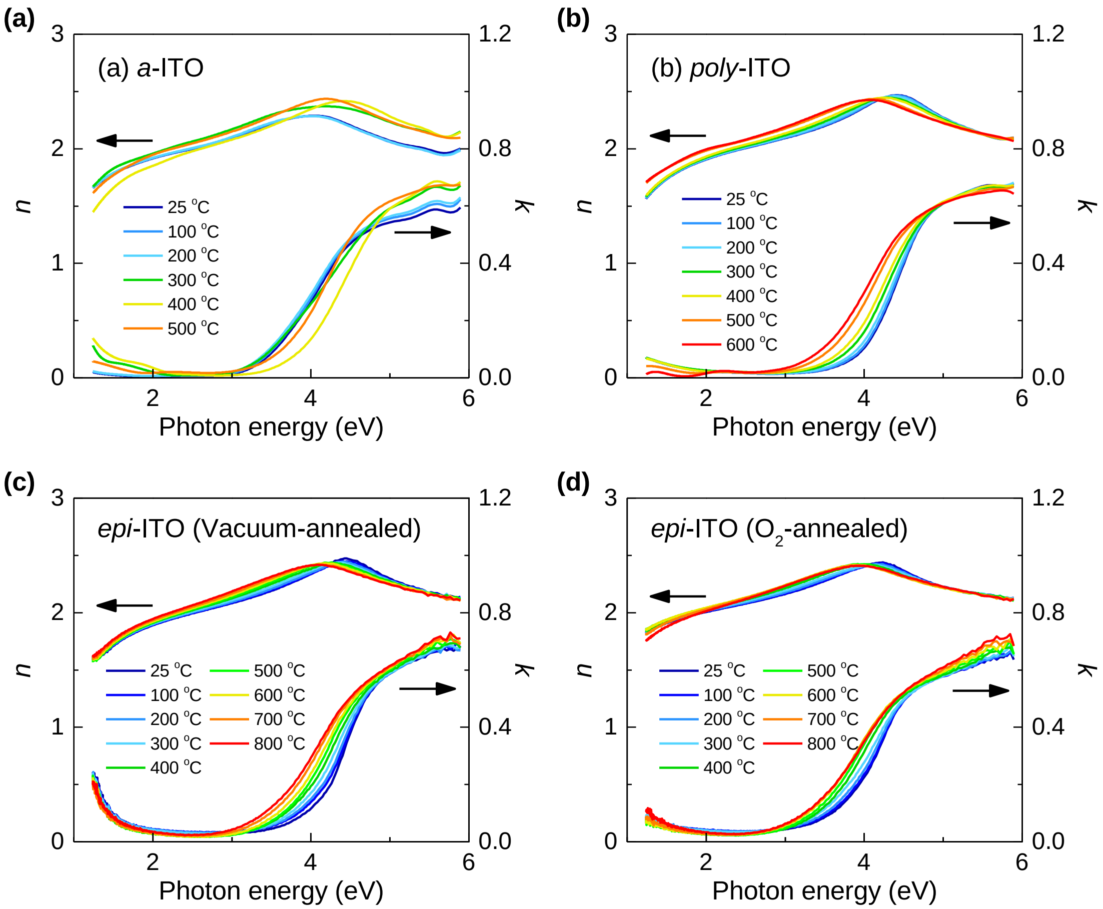

Figure S5. The refractive index (*n*) and extinction coefficient (*k*) spectra of (a) *a*-ITO, (b) *poly*-ITO and *epi*-ITO thin films after annealing in (c) the vacuum and (d) O_2 environments at various temperatures.

Table S1. Sample deposition methods, growth, and post-annealing conditions for *a*-ITO, *poly*-ITO, and *epi*-ITO thin films.

	a-ITO	*poly*-ITO	*epi*-ITO	
			Vacuum-annealed	O_2-annealed
Deposition methods	*dc*-Sputtering	*dc*-Sputtering	PLD	PLD
Growth conditions	T = 100 ℃<br>$p(O_2)$ = 0.1 mTorr<br>Ar: 100 sccm<br>O_2: 2 sccm	T = 500 ℃<br>$p(O_2)$ = 0.1 mTorr<br>Ar: 100 sccm<br>O_2: 2 sccm	T = 600 ℃<br>$p(O_2)$ = 10 mTorr	T = 600 ℃<br>$p(O_2)$ = 10 mTorr
Post-annealing conditions	-	T = 500 ℃<br>$p(O_2)$ = 10 mTorr	T = 800 ℃<br>$p(O_2) < 0.001$ mTorr	T = 800 ℃<br>$p(O_2)$ = 10 mTorr

References

1. Johs, B. D., French, R. H., Kalk, F. D., McGahan, W. A. & Woollam, J. A. Optical analysis of complex multilayer structures using multiple data types. *Proceedings SPIE* **2253**, 1098-1106 (1994).

2. Bruggeman, D. A. G. Berechnung verschiedener physikalischer Konstanten von heterogenen Substanzen. I. Dielektrizitätskonstanten und Leitfähigkeiten der Mischkörper aus isotropen Substanzen. *Ann. Phys.* **416**, 636-664 (1935).
